# Microenvironment-responsive nanosystems for ischemic stroke therapy

**DOI:** 10.7150/thno.99822

**Published:** 2024-09-03

**Authors:** Fang Wu, Zhijian Zhang, Shengnan Ma, Yanyan He, Yuxi He, Lixia Ma, Ningjing Lei, Wenjing Deng, Fazhan Wang

**Affiliations:** 1 Department of Neuro-Intensive Care Unit, the First Affiliated Hospital of Zhengzhou University, Zhengzhou University, Zhengzhou, 450052, Henan, China.; 2 Department of Immunology, School of Basic Medical Sciences, Zhengzhou University, Zhengzhou, 450001, China.; 3 Medical Research Center, the First Affiliated Hospital of Zhengzhou University, Zhengzhou University, Zhengzhou, 450052, Henan, China.; 4 Department of Endocrinology and Metabolism, the First Affiliated Hospital of Zhengzhou University, Zhengzhou University, Zhengzhou, 450052, Henan, China.; 5 Henan Key Laboratory of Chronic Disease Prevention and Therapy & Intelligent Health Management, Zhengzhou, 450052, Henan, China.

**Keywords:** Ischemic stroke, Ischemic cerebral microenvironment, Stimuli-responsive materials, Nanosystems, Brain-targeted drug delivery

## Abstract

Ischemic stroke, a common neurological disorder caused by impaired blood supply to the brain, presents a therapeutic challenge. Conventional treatments like thrombolysis and neuroprotection drugs lack ideal drug delivery systems, limiting their effectiveness. Selectively delivering therapies to the ischemic cerebral tissue holds great potential for preventing and/or treating ischemia-related pathological symptoms. The unique pathological microenvironment of the brain after ischemic stroke, characterized by hypoxia, acidity, and inflammation, offers new possibilities for targeted drug delivery. Pathological microenvironment-responsive nanosystems, extensively investigated in tumors with hypoxia-responsive systems as an example, could also respond to the ischemic cerebral microenvironment and achieve brain-targeted drug delivery and release. These emerging nanosystems are gaining traction for ischemic stroke treatment. In this review, we expound on the cerebral pathological microenvironment and clinical treatment strategies of ischemic stroke, highlight various stimulus-responsive materials employed in constructing ischemic stroke microenvironment-responsive nano delivery systems, and discuss the application of these microenvironment-responsive nanosystems in microenvironment regulation for ischemic stroke treatment.

## 1. Introduction

Stroke, a devastating condition affecting survivors' quality of life worldwide, lacks effective therapeutic options. Studies report that approximately 80% of all stroke cases are ischemic 1. Ischemic stroke occurs when a blockage in blood vessels deprives a localized area of the brain of oxygen and blood supply, leading to ischemic damage or necrosis of brain cells. During ischemic injury, hypoxia and ischemia results in abnormal brain cell metabolism. This abnormal metabolism involves a series of biochemical events, including adaptive changes in metabolism, cell death, and the triggering of a cascade within the ischemic brain tissue. This cascade includes the overproduction of reactive oxygen species (ROS), decreased pH, inflammation, and disruption of the blood-brain barrier (BBB). The complexity of the ischemic pathological microenvironment in ischemic brain tissue is further compounded by its dynamic changes as the disease progresses, potentially limiting the efficacy of current stroke therapies.

Ischemic stroke treatment hinges on immediate recanalization of the blocked artery, as disability and mortality rise with time of obstruction. The gold standard involves intravenous thrombolysis or mechanical thrombectomy to achieve early revascularization and restore blood flow to the brain 2. Recombinant tissue plasminogen activator (tPA) is the only Food and Drug Administration-approved thrombolytic therapy for ischemic stroke 3. While tPA has significantly improved stroke treatment and patient quality of life, its clinical application faces limitations. These include the risk of hemorrhagic transformation and a narrow therapeutic window due to high dosage, short half-life, and poor targeting of tPA itself. Additionally, endovascular mechanical thrombectomy, commonly used for large vessel occlusions 4, is only suitable for a limited number of patients 5. Additionally, reperfusion injury, an exacerbation of brain tissue damage caused by reoxygenation after artery recanalization, complicates the picture. The complex and dynamic ischemic cascade, coupled with the low selectivity of drugs for the ischemic penumbra, likely contributes to the lack of truly effective treatments for ischemic stroke. Despite ongoing research on novel therapeutic approaches to overcome these limitations, success has been limited. Therefore, the development of novel pharmacotherapies with improved brain penetration and controlled drug release within ischemic brain tissue is crucial for enhancing the therapeutic effect against ischemic stroke.

Nanoscale drug delivery systems (nDDS), particularly stimuli-responsive nanosystems, offer several unique advantages for targeted drug delivery and controlled release. These systems are designed to release drugs in specific locations in response to specific stimuli such as hypoxia, low pH, and elevated ROS levels. This targeted approach allows them to avoid drug leakage in circulation, preserve the activity of bioactive compounds, and minimize side effects. Stimuli-responsive nanosystems responsive to tumor microenvironment cues like hypoxia, low pH, upregulated ROS levels, and specific enzymes have been explored in cancer treatment for chemotherapy, gene therapy, and immunotherapy 6, 7. Inspired by these successes, researchers have constructed microenvironment-responsive nanosystems for ischemic stroke treatment. These hypoxia, low pH, high ROS, or enzyme-responsive nanosystems exhibit smart drug delivery and controlled release within ischemic brain regions, demonstrating promising therapeutic efficacy in recent years.

**Figure 1:**
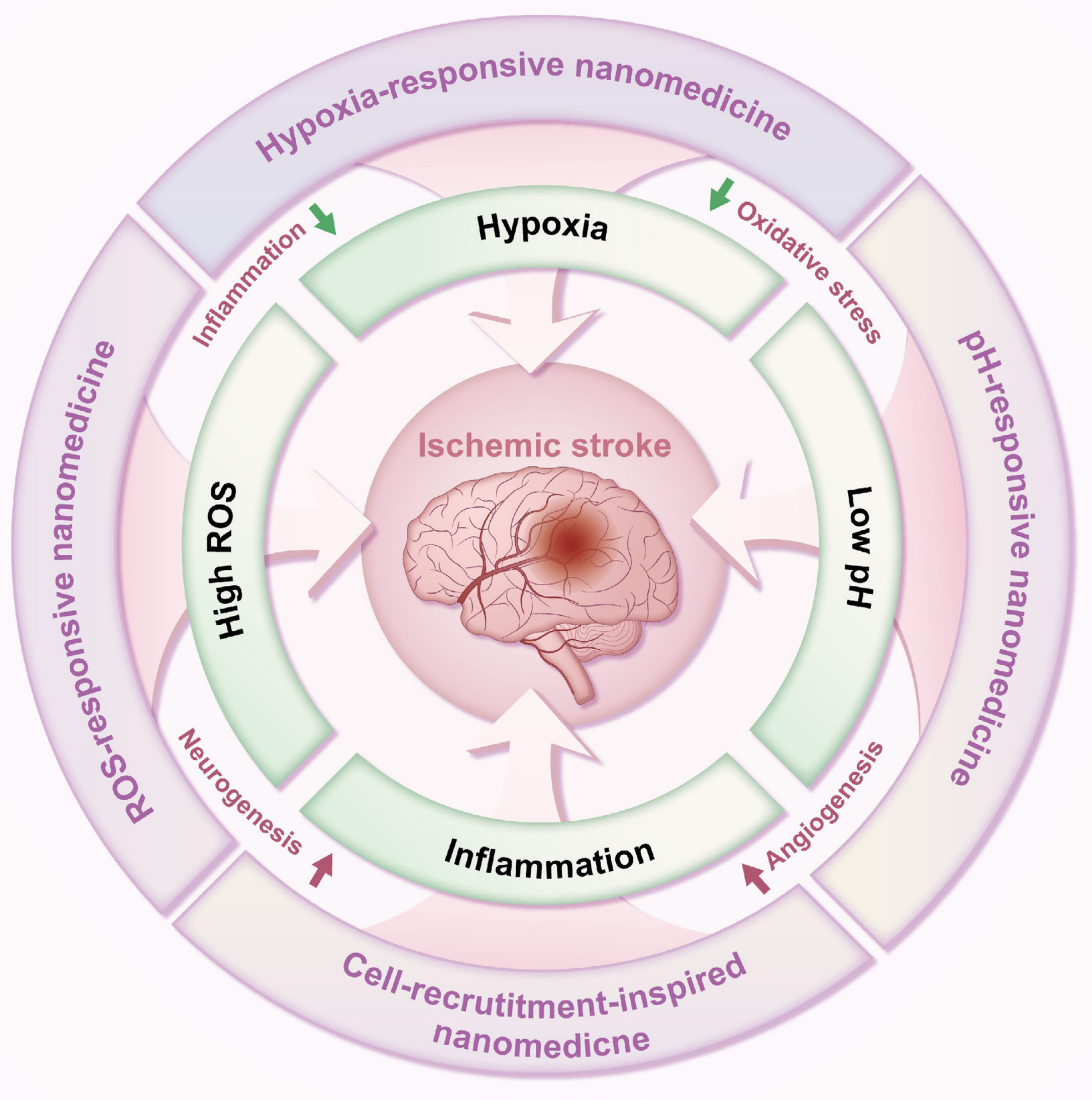
**Schematic diagram of microenvironment-responsive nanomedicine for ischemic stroke treatment.** Stimuli-responsive nDDS can exert enhanced delivery and controlled release of therapeutic drugs in response to specific local pathophysiological stimuli (e.g., hypoxia, high ROS levels, inflammation, and low pH) in the ischemic brain tissue for ischemic stroke treatment through promoting angiogenesis, boosting neurogenesis, attenuating oxidative stress, or mitigating inflammation.

In light of the complex pathological brain microenvironments following ischemic stroke, designing nano-delivery systems responsive to these environments presents a novel therapeutic strategy. Nanomedicines have emerged as promising platforms for ischemic stroke therapy. As a result, there have been numerous reviews that explore this rapidly expanding field 8-14. Different from previous reviews, this review describes the pathological brain microenvironments and current treatment options for ischemic stroke. We further highlight the various stimuli-responsive elements employed in constructing these ischemic stroke microenvironment-responsive nano-delivery systems. Finally, the application of such microenvironment-responsive systems for ischemic stroke treatment is summarized, focusing on their response to hypoxia, elevated ROS, inflammation, and low pH within the brain microenvironment **(Figure 1),** providing reference value for developing precision medicines against ischemic stroke.

## 2. Pathophysiological microenvironments of ischemic stroke

Compared to normal brain tissue, ischemic cerebral tissue develops a unique pathophysiological microenvironment following stroke. This microenvironment is characterized by hypoxia, high levels of ROS, inflammation, acidosis, BBB disruption, and overexpression of certain enzymes. These changes are primarily caused by reduced oxygen and glucose supply due to decreased cerebral blood flow 15. The ischemic cerebral microenvironment not only serves as a hallmark of stroke but also actively participates in stroke progression. Therefore, it has become a crucial target for ischemic stroke treatment and a widely used stimulus for controlling drug delivery and release. A comprehensive understanding of this pathological microenvironment is critical for developing stimuli-sensitive nDDS for ischemic stroke treatment. This section will elucidate the pathological features and formation mechanisms of specific aspects of the cerebral microenvironment after ischemic stroke, including hypoxia, high ROS levels, inflammation, decreased pH, and a disturbed BBB.

### 2.1. Hypoxia

Hypoxia, a defining characteristic of the ischemic stroke microenvironment* in vivo*, plays a critical role in disease progression and treatment failure. Oxygen and essential nutrients are crucial for brain cell respiration and metabolism. It has been reported that interstitial partial pressure of oxygen rapidly decreased to approximately 4% in the core and 30% of preischemic values in the penumbra in a rat cerebral ischemia model 16. Following cerebral blood flow interruption, neurons in ischemic brain regions experience insufficient oxygen and nutrient supply, leading to hypoxia and, ultimately, irreversible necrosis. To adapt to this hypoxic stress, brain cells attempt to conserve energy by hindering their metabolic activity. This alters the biochemical environment surrounding brain cells and disrupts cellular processes, including metabolism. Nanomedicines that directly deliver oxygen to the ischemic lesion 17, 18 or promote hydrogen peroxide (H_2_O_2)_ decomposition to produce oxygen in situ to alleviate the hostile hypoxic environment have demonstrated their efficacy for ischemic stroke therapy 19, 20. Interestingly, the hypoxic microenvironment of ischemic stroke shares similarities with that of tumors, which holds great promise for the development of diagnostic tools 21, targeted drug delivery systems 22, and controlled drug release 23 strategies specific to cerebral ischemic lesions.

### 2.2. Increased oxidative stress

Oxidative stress is a key hallmark of ischemic stroke. The excessive accumulation of ROS, such as H_2_O_2_, following cerebral ischemia onset triggers oxidative stress, which further exacerbates neuronal death 24. Changes in metabolic patterns within ischemic brain cells facilitate ROS accumulation due to mitochondrial dysfunction. This dysfunction results in a double-edged sword effect: it enhances the activity of ROS-producing enzymes while simultaneously decreasing the activity of antioxidant enzymes. This imbalance between ROS generation and scavenging leads to their substantial accumulation 25. Elevations in H_2_O_2_ were detected during ischemia and reperfusion with an extracellular H_2_O_2_ concentration of 160 μM in ischemic brain, while 10 μM H_2_O_2_ concentration was observed in the normal condition of brain 26, 27. Excessive ROS in the ischemic region can react with lipids, proteins, and DNA, leading to inflammation, cell dysfunction, and, ultimately, cell death. Furthermore, oxidative stress from ROS disrupts the expression of key tight junction (TJ) proteins in brain vascular endothelial cells, thereby impairing the functional integrity of the BBB 28. Therefore, upregulated ROS in the ischemic brain region represents not only a therapeutic target but also a potential smart trigger for controlled drug release. By utilizing ROS as a trigger, drug delivery systems can achieve enhanced local concentrations of therapeutic drugs at ischemic sites.

### 2.3. Inflammation

The inflammatory process evolves in different stages in stroke 29. In the acute phase (first hours after stroke onset), microglia/macrophages clear the necrotized cells and the first entry of leukocytes to the ischemic brain tissue has been described. During subacute phase (the first days after the ischemic insult), a resolution of the inflammatory process was observed. In the late stage, the inflammatory cells contribute to astrocytic and microglial reparatory processes. Inflammation is a critical pathological feature affecting the progression and treatment of ischemic stroke. Following cerebral ischemia, apoptotic or necrotic cells release various danger-associated molecular patterns 30. These danger-associated molecular patterns stimulate the accumulation of inflammatory cytokines and chemokines, such as tumor necrosis factor-α, interleukin-6, and monocyte chemotactic protein. The inflammatory cytokines activate microglia and mediate their differentiation into the pro-inflammatory M1 phenotype 31. Excessive activation of M1 microglia leads to the release of many pro-inflammatory factors, including inducible nitric oxide synthase and ROS, which exacerbate brain damage and trigger an inflammatory cascade reaction 32. Furthermore, these inflammatory mediators can recruit peripheral immune cells such as neutrophils, monocytes, and macrophages to the damaged brain areas. This recruitment can lead to microvascular occlusion and further exacerbate the release of oxygen-free radicals and cytotoxic substances, ultimately causing neuronal damage and perpetuating a vicious cycle of inflammation. Peripheral neutrophils, monocytes/macrophages are recruited into the ischemic brain and transform into macrophages after stroke, which have been implicated in stroke-induced inflammation and injury 33. Regulation of the migration ability of monocytes/macrophages 34 from periphery to ischemic brain issues or modulation of the polarization 35 of monocytes/macrophages before their migration to brain tissue have demonstrated their potential for treating ischemic stroke. The inflammatory response associated with peripheral immune cell recruitment to the ischemic brain tissue 33 might present an opportunity for the development of inflammation-responsive nanomedicines for ischemic stroke. If peripheral neutrophils, monocytes, or macrophages could be modulated prior to their migration to brain tissue, the inflammatory response associated with peripheral immune cell recruitment might present an opportunity for the development of inflammation-responsive nanomedicines for ischemic stroke.

### 2.4. Low pH

Ischemic brain tissue exhibits a mildly acidic environment, characterized by decreased pH compared to healthy tissue. This phenomenon is primarily due to disrupted energy metabolism following ischemia. The switch from aerobic metabolism to anaerobic glycolysis leads to the accumulation of lactic acid and pyruvate, both of which contribute to a decrease in pH 36. Additionally, the hydrolysis of excessive adenosine triphosphate in ischemic stroke generates hydrogen ions, further acidifying the brain tissue surrounding the ischemic core. Studies have shown that the pH value in this region rapidly decreases to 6.77 within 1 hour after cerebral ischemia and continues to decline to approximately 6.5 after 4 hours 37. Moreover, tissue pH in the ischemic core may become as low as pH 6.0, while tissue pH fluctuates around pH 6.5-6.9 in the peri-infarct penumbra 36. This pH gradient between ischemic and normal brain tissue offers a promising microenvironment for targeted drug delivery and controlled release of therapeutics specifically to the ischemic area.

### 2.5. Pathological features of the BBB

The BBB is a critical semipermeable membrane separating the central nervous system from the peripheral circulation. Under normal physiological conditions, the BBB strictly regulates permeability through TJ protein complexes (including claudin and occludin), effectively preventing the entry of peripheral blood cells and macromolecules into the brain 38. However, ischemic stroke disrupts this tightly regulated system. Matrix metalloproteinases released after ischemia can damage the TJ between endothelial cells, disrupting the BBB 39. This compromised BBB exhibits increased permeability, causing dyshomeostasis of ions and water, ultimately leading to cerebral edema 40. Furthermore, the disruption of BBB function allows the infiltration of various immune cells and macromolecules into the ischemic brain, further exacerbating the existing BBB damage 41. This disruption of BBB presents an opportunity for targeted drug delivery, though obstacles of BBB against therapeutic drugs for ischemic stroke treatment. By leveraging the altered permeability of the BBB during ischemic stroke, researchers have designed various nanosystems 42-47 that can specifically access the ischemic site, overcoming the normally formidable barrier that the BBB presents for drug delivery to the brain.

## 3. Current strategies against ischemic stroke

Timely and effective interventions are crucial for improving the prognosis of patients with ischemic stroke. Current treatment strategies primarily focus on two main goals: restoring blood flow through vascular recanalization and minimizing neuronal damage caused by ischemia. Vascular recanalization therapies can be broadly categorized into two approaches: thrombolytic drugs and endovascular therapy. This section will elucidate the current strategies for vascular recanalization and neuroprotection, as well as summarize the clinical trials involving nanomedicines for ischemic stroke treatment.

### 3.1. Restoration of cerebral blood flow

The primary clinical goal for ischemic stroke treatment is the immediate restoration of blood flow within the ischemic cerebral tissue. Current strategies for achieving this revascularization focus on eliminating or reducing blood clots through either mechanical thrombectomy or pharmacological thrombolysis. Intravenous administration of tPA remains the only Food and Drug Administration-approved medication for ischemic stroke; however, its clinical application is limited by a narrow therapeutic window (less than 4.5 hours after stroke onset) and an increased risk of intracerebral hemorrhage (approximately 6%) 48. Endovascular mechanical thrombectomy offers a complementary approach to thrombolysis for restoring blood flow, particularly in cases of large vessel occlusions. While this surgical intervention has become the standard treatment for such patients, its suitability is restricted to a limited population. The superiority of mechanical thrombectomy in minor stroke is not proven, though 6 randomized controlled trials have demonstrated the superiority of mechanical thrombectomy in patients with acute ischemic stroke caused by occlusion of arteries of the proximal anterior circulation 49. Moreover, various factors such as the amount of time a thrombus formation, proportion of fibrin relative to the red blood cells and the content of white blood cells in the thrombus determine its susceptibility to mechanical thrombectomy 50. Importantly, thrombolytic drugs carry the risk of bleeding complications due to their activation of fibrinolysis in non-ischemic areas. Therefore, there remains a significant challenge in the field of revascularization therapy. Some of targeted and controlled therapeutic delivery systems demonstrate the efficacy in ischemic stroke treatment by promoting angiogenesis 51, 52, indicating the great promise of emerging delivery systems for ischemic stroke treatment by restoration of cerebral blood flow.

### 3.2. Neuroprotection

Ischemic stroke triggers a neuroinflammatory response characterized by the production of pro-inflammatory cytokines and ROS, which ultimately leads to irreversible cell death. Neuroprotection, therefore, emerges as a promising therapeutic strategy aimed at enhancing neuronal survival following ischemic stroke. By promoting neuronal survival, this approach can potentially expand the therapeutic window for drug intervention and facilitate neurological function repair and recovery 53. Current neuroprotective agents, such as free radical scavengers, calcium channel blockers, γ-aminobutyric acid agonists, nitric oxide antagonists, and glutamate antagonists, primarily offer symptomatic relief. Unfortunately, no available therapy can definitively prevent neuronal tissue damage after ischemic stroke. Novel strategies are urgently needed to target the underlying causes of the disease, thereby suppressing neuronal death and the associated destructive molecular events. Over the past two decades, a significant number of neuroprotective agents have been developed, with approximately 10% reaching clinical trials. Disappointingly, none of these agents have achieved convincing efficacy for clinical approval despite demonstrably positive results in animal models 54. The low BBB permeability and short half-lives of these neuroprotectants likely contribute to the failures observed in clinical trials. Novel approaches 46, 55, 56 to enhance the accumulation of neuroprotectants within the ischemic site hold promise for improving therapeutic efficacy and facilitating successful clinical translation. Stimuli-responsive drug delivery systems, a recent advancement in drug delivery technology, offer a potential solution for targeted and controlled delivery of neuroprotective agents to ischemic brain tissue.

## 4. Stimulus-responsive elements for ischemic stroke microenvironment-responsive nanosystems

Stimuli-responsive nanosystems have emerged as a promising approach for targeted drug delivery in recent years 57. These nanomedicines are engineered to release their therapeutic cargo in a controlled manner at the desired site upon exposure to specific internal or external stimuli, such as hypoxia, lower pH, elevated ROS levels, and ultrasound 58. Pathological microenvironment-responsive nanomedicines offer significant advantages compared to externally triggered systems, as they do not require additional equipment for drug release activation. The distinct pathological features of the ischemic hemisphere, including hypoxia, high ROS levels, inflammation, and low pH, provide a unique opportunity to exploit stimuli-responsive nanomedicines for ischemic stroke treatment. This review focuses on the pathological microenvironment-responsive elements and nanomedicines designed for ischemic stroke therapy. For instance, hypoxia-responsive nanomedicines leverage hypoxia-responsive chemical components that undergo structural changes upon encountering the hypoxic microenvironment, leading to the disassembly of the nanocarriers and the release of the encapsulated drugs. This section will delve into the structure and stimuli-responsive mechanisms of hypoxia-sensitive, ROS-sensitive, inflammation-sensitive, and low pH-sensitive elements for ischemic stroke microenvironment-responsive drug delivery systems.

### 4.1. Hypoxia-sensitive elements

Hypoxia, a hallmark pathological feature of various diseases, including stroke, presents a unique target for stimuli-responsive drug delivery systems due to its rarity and distinctiveness in healthy tissues. Hypoxia-sensitive nanomedicines or hypoxia-activated prodrugs comprise a class of inactive prodrugs that require the specific hypoxic environment of a lesion site (such as ischemic stroke, tumors 59, or other internal hypoxic regions) to trigger controlled drug release. These unique properties stem from hypoxia-responsive chemical bonds incorporated into the nanomedicines, including nitroaromatic 60, 61, azobenzene 62, 63, and nitrobenzyl alcohol 64, 65, which undergo structural changes upon exposure to a hypoxic environment (**Table 1**). Nitroimidazole was the first functional group employed in the synthesis of hypoxia-responsive polymers. Under anaerobic conditions, nitroimidazoles can be reduced to aminoimidazoles by cellular enzymes, leading to an alteration in their hydrophilicity and hydrophobicity. This change in polarity disrupts the self-assembly of the nitroimidazole-containing nanocarriers, resulting in the release of the encapsulated drug 66. Azobenzene represents another widely utilized hypoxia-responsive moiety. Like nitroimidazoles, azobenzenes readily accept electrons and undergo reduction reactions in low-oxygen environments. This reduction cleaves the azo structure, generating aniline derivatives 67. While a variety of hypoxia-responsive chemical bonds are currently being explored in the laboratory, the development of novel hypoxia-responsive structures with enhanced controllability and safety profiles is crucial to meet future clinical needs and advance the field of hypoxia-responsive nanomedicines for stroke treatment.

### 4.2. ROS-sensitive elements

ROS-responsive nanomedicines have been designed to exploit the elevated ROS levels within the ischemic brain microenvironment for enhanced and controlled drug delivery. Researchers have explored a variety of ROS-responsive materials for use in drug delivery systems, including those containing sulfur, selenium, boric acid, proline oligomers, and carbonyls incorporating selenium/tellurium, thioether, thioketal, ferrocene, aminoacrylate, peroxalate ester, boronic ester, and polyproline (**Table 2**). The reaction mechanisms of these ROS-responsive materials are summarized in the referenced table. The primary mechanisms for ROS-triggered therapeutic release involve cleavage-induced carrier degradation, solubility changes leading to carrier disassembly, and cleavage of the carrier-drug linker 68. Boronic esters, for instance, are frequently employed due to their susceptibility to cleavage by ROS. In the presence of elevated ROS levels within the ischemic brain microenvironment, the cleavage of boronic esters results in the controlled release of drugs, leading to significantly higher drug accumulation than non-degradable counterparts 69. Notably, the terms "thioketal linkers" and "aminoacrylate which cleaved by ROS oxidants" are tautologous; both describe linkers cleaved by ROS. Furthermore, thioether-containing polymers exhibit a transition from hydrophobic to hydrophilic states in response to highly oxidative environments 70, 71. Poly(alkylene sulfide)s, for example, undergo oxidation to poly(alkylene sulfoxide) and ultimately poly(alkylene sulfone), with a corresponding shift from hydrophobic to hydrophilic character 72. Similarly, sulfur-containing polymers and tellurium compounds can be oxidized by ROS, leading to a transition from hydrophobic to hydrophilic states. These properties have also been harnessed in the design of ROS-responsive nanocarriers 73. A detailed discussion of the synthesis and oxidation properties of other ROS-responsive nanomaterials can be found in previous reviews 74. Therefore, this section will focus on the progress and applications of stimuli-responsive drug delivery systems.

### 4.3. Inflammation-sensitive elements

The inflammatory response within the ischemic brain microenvironment triggers the recruitment of immune cells, including leukocytes, monocytes/macrophages (predominantly polymorphonuclear leukocytes or neutrophils), mesenchymal stem cells (MSCs), and neural stem cells (NSCs), to the ischemic site 95. This phenomenon has led to the development of strategies that leverage inflammation-induced cell recruitment for enhanced drug delivery and accumulation within the inflamed brain tissue while minimizing off-target drug release (**Table 3**). Neutrophils, the first responders to tissue injury, rapidly migrate to the ischemic region. Neutrophil-based nDDS have been explored as a promising approach to facilitate targeted therapeutic delivery to ischemic areas 96. Similarly, monocytes/macrophages retain inflammatory-oriented chemotactic abilities driven by chemokines and adhesion molecules upregulated during ischemic stroke. Nanoparticles coated with monocytes/macrophage membranes demonstrate excellent targeting capability to the inflamed brain tissue. The ischemia-homing potential of NSCs suggests their membrane could also be investigated for use in drug delivery systems 97. Platelets, which play a role in responding to inflammation and repairing vascular damage, have inspired the development of platelet membrane-encapsulated nanoparticles for targeted therapeutic delivery to the injury site 98. Furthermore, inspired by the ability of malignant tumor cells to readily permeate the BBB during brain metastasis, tumor cell membrane-based nanomedicines have been constructed for drug delivery to inflamed ischemic regions 99, 100. Bacteria or bacterial-derived outer membrane vesicles have also been explored as platforms for nanomedicine development to enhance drug delivery to inflamed ischemic brain tissue 101. These biomimetic nanosystems, triggered by inflammation-based cell recruitment, exhibit inherent tropism to the damaged ischemic tissue 102, 103. This approach offers several advantages over traditional nanoparticles, including improved permeability across the BBB and the potential to reduce the infiltration of peripheral inflammatory cells into the ischemic region. Despite significant advancements in inflammation-related cell recruitment-based biomimetic nanosystems, further research ssociated with reproducibility and scalability of these nanosystems is urgently needed to fully exploit the capabilities of these systems for ischemic stroke treatment.

### 4.4. pH-sensitive delivery elements systems

The pH gradient between ischemic and healthy brain tissue has spurred the development of pH-responsive nDDS for targeted drug release within ischemic regions. These systems offer the potential for improved therapeutic efficacy and reduced side effects. Low pH within the ischemic environment can trigger structural changes in pH-responsive amphiphilic polymer-based drug carriers, resulting in decomposition and subsequent drug release (**Table 4**). Two primary strategies exist for constructing pH-responsive nanocarriers. The first approach incorporates pH-responsive elements such as amino, phosphoric acid, and carboxyl groups. Under physiological conditions (normal pH), these groups remain electrically neutral, thereby maintaining the stability of the nanocarrier. However, upon exposure to the acidic environment of ischemic tissue, these groups become protonated, leading to electrostatic repulsion and, ultimately, the dissolution of the nanocarrier and release of the encapsulated drug 111. The second approach utilizes acid-sensitive chemical bonds, including imine bonds, hydrazone bonds, β-Carboxyamide bonds, ketals, and acetals, within the nanocarrier structure. These bonds are susceptible to hydrolysis and cleavage under acidic conditions, resulting in the disassembly of the nanocarrier and controlled release of the drug 112. While other acid-labile linkers, such as acetals, have also been explored for pH-responsive nanomedicine design, a comprehensive discussion of these is beyond the scope of this review.

## 5. Application of ischemic stroke microenvironment-sensitive delivery systems

Ischemic stroke presents a complex and dynamic pathophysiological microenvironment, highlighting the significance of smart nDDS for the "on-demand" release of therapeutic agents. These systems offer distinct advantages by preventing drug elimination, enabling targeted delivery, and facilitating controlled release of therapeutics to the desired area. Stimuli-responsive nDDS hold great promise, as they can trigger enhanced delivery and controlled release of drugs in response to specific local pathophysiological stimuli (e.g., hypoxia, high ROS levels, inflammation, and low pH) unique to the ischemic brain tissue. Extensive research has demonstrated the potential of stimuli-responsive nanomedicines to significantly improve therapeutic outcomes for ischemic stroke by enabling controlled drug delivery and release at the site of injury while minimizing adverse effects. This section summarizes studies on stimuli-responsive nDDS for ischemic stroke treatment, focusing on their potential to promote angiogenesis, decrease oxidative stress, mitigate inflammation, and improve neuronal apoptosis - all of which contribute to functional recovery after ischemic stroke (**Table 5**).

### 5.1. Hypoxia-sensitive delivery systems

Ischemic stroke, characterized by vascular occlusion, triggers the formation of a hypoxic microenvironment within the lesioned brain tissue. This hypoxic environment not only exacerbates tissue damage but also hinders the efficacy of traditional therapeutic approaches. Notably, hypoxia is a significant pathophysiological hallmark not only of ischemic stroke 164 but also of various other diseases, including solid tumors 165, 166 and inflammatory diseases 167. Hypoxia-responsive nanomedicines can specifically target and release drugs within hypoxic tumor regions through well-defined response mechanisms, leading to improved treatment efficacy and reduced damage to healthy tissues. While the application of hypoxia-responsive nanomedicines in the field of oncology has reached a more mature stage, their use in ischemic stroke treatment remains in its early stages. Nevertheless, the advancements made in hypoxia-responsive anti-tumor nanomedicine can provide valuable insights for the development of similar strategies for ischemic stroke.

A recent study investigated a novel hypoxia-responsive calixarene-grafted peptide specifically designed for ischemic stroke treatment by leveraging the hypoxic microenvironment within the ischemic zone 129. The researchers employed a click reaction to conjugate methacrylate-functionalized calixarene with a thiol-containing Q11 peptide. Subsequently, a phosphate-buffered saline (PBS)-induced β-sheet formation process yielded the hypoxia-responsive hydrogel. Notably, the calixarene formed a stable complex with fingolimod (FTY720). However, under hypoxic conditions, cleavage of the calixarene's azo groups triggered the decomplexation of FTY720, resulting in hypoxia-responsive drug release. The *in vitro* release profile of FTY720 from the self-assembled peptide hydrogel was evaluated using rat liver microsomes with reduced nicotinamide adenine dinucleotide phosphate, sodium dithionite (SDT) as a reductase mimetic, and an oxygen-glucose deprivation model. The biological evaluation demonstrated that the released FTY720 alleviated the inflammatory response following stroke by promoting a shift from M1 to M2 macrophages. Furthermore, the hypoxia-responsive peptide hydrogel reduced infarct volume, protected local neurons, inhibited cell apoptosis, and promoted motor function recovery.

**Figure 2:**
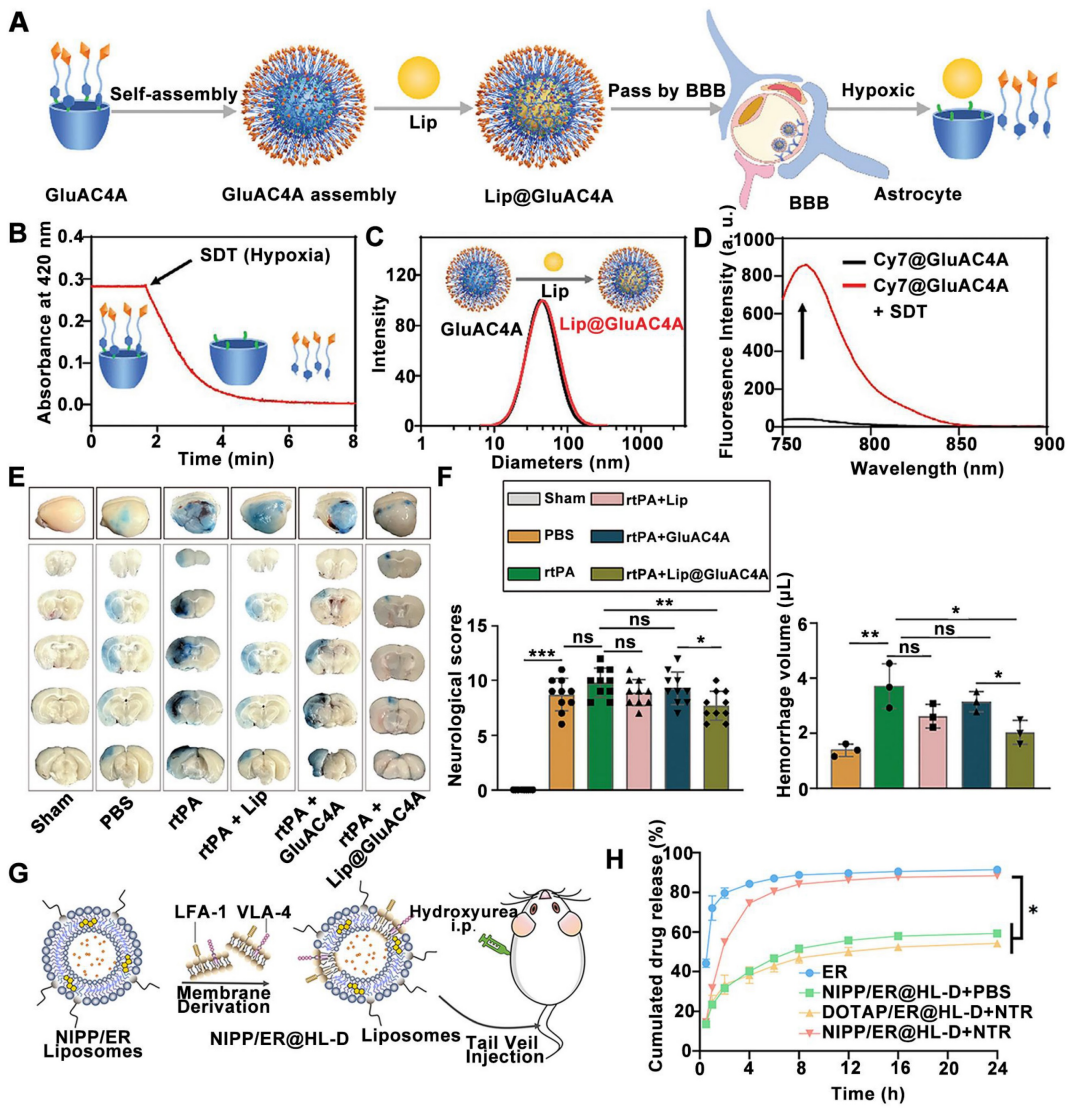
** Hypoxia-sensitive nanomedicines for ischemic stroke treatment by controlled drug release upon hypoxia trigger in brain microenvironment.** (A) Schematic illustration of Lip@GluAC4A and its hypoxia responsiveness release of Lip. (B) Dynamic absorbance of GluAC4A at 420 nm following the addition of SDT in PBS. (C) Particle size of Lip@GluAC4A and GluAC4A measured by Dynamic Light Scattering. (D) The release of Cy7@GluAC4A recorded by fluorescence before and after the addition of SDT in PBS. (E) Representative images of Evans blue extravasation 24 h after MCAO in mice. (F) Quantification of neurological deficits by neurological severity score and cerebral hemorrhage volume using the Drabkin reagent. Adapted with permission from 131, copyright 2024 John Wiley and Sons. (G) Schematic diagram of neutrophil-like cell membrane fusion hypoxia-sensitive liposomes for the treatment of hyperacute ischemic stroke. (H) *In vitro* release profiles of ER in PBS 7.4 at 37 °C under the simulated hypoxic conditions (10 μg/ mL Nitroreductase (NTR) + 100 μM nicotinamide adenine dinucleotide phosphate in PBS) or in PBS (n = 3). Adapted with permission from 130, copyright 2023 Royal Society of Chemistry.

Wu *et al.* recently described the design of a novel macrocyclic carrier for the targeted delivery of liproxstatin-1 (Lip), a ferroptosis inhibitor, to the ischemic site (**Figure 2A**). This system utilizes glucose-modified azocalixarene (GluAC4A) to achieve targeted delivery and ameliorate hemorrhagic transformation induced by recombinant tPAtt 131. The responsiveness of GluAC4A to hypoxia, a key feature of the ischemic microenvironment, was investigated using SDT, a chemical mimic of azoreductase. Treatment with SDT resulted in a continuous decrease in the absorbance of GluAC4A at 420 nm, as monitored by ultraviolet-visible spectroscopy, indicating the reduction of azo groups within GluAC4A (**Figure 2B**). Self-assembly of GluAC4A yielded nanoparticles (**Figure 2C**). The release profiles of Cy7-loaded GluAC4A (Cy7@GluAC4A) before and after SDT treatment demonstrated the hypoxia-responsive release of therapeutics encapsulated within GluAC4A (**Figure 2D**). Finally, the effectiveness of the supramolecular carrier GluAC4A in mitigating rtPA-induced side effects was investigated in a middle cerebral artery occlusion (MCAO) mouse model. Evans blue extravasation, a marker of BBB permeability, was remarkably improved in mice treated with rtPA + Lip@GluAC4A compared to those treated with rtPA + GluAC4A alone (**Figure 2E**). Furthermore, treatment with rtPA + Lip@GluAC4A significantly reduced neurological severity scores and brain hemorrhage compared to treatment with rtPA + GluAC4A alone (**Figure 2F**). These findings suggest that Lip@GluAC4A offers a protective effect on the BBB after ischemic stroke *in vivo*.

Besides azocalixarene, 2-nitroimidazole derivatives have been employed to design hypoxia-sensitive liposomes. These liposomes incorporate a fusogenic neutrophil-like cell membrane and the neuroprotective agent edaravone as bioactive components for ischemic stroke rescue therapy 130. Similar with other hypoxia-responsive nDDS designed for tumor treatment, hypoxia-sensitive liposomes for ischemic stroke share common lipid components but include a key component that responds to the hypoxic microenvironment. To achieve targeted delivery of stroke therapeutics to the ischemic penumbra following stroke, the researchers formulated hypoxia-sensitive NIPP liposomes by combining their hypoxia-responsive lipid (2-nitroimidazole lipid derivative) with cholesterol, soybean lecithin, DSPE-PEG_2000_, and Tween-80. NIPP liposomes were further fused with cell membrane of differentiated HL-60 cells (highly express lymphocyte function associated antigen-1 (LFA-1) and very late antigen-4 (VLA-4)) to target inflamed brain microvascular endothelial cell (**Figure 2G**). Mechanistic studies of hypoxia-sensitive release revealed that the hydrophobic 2-nitroimidazole moiety within the hypoxia-sensitive lipid undergoes a transformation to hydrophilic 2-aminoimidazoles under hypoxic conditions. Hypoxia--responsive release properties of NIPP / Edaravone (ER)@HL-D liposomes were assessed by an *in vitro* release assay. The ER release from NIPP/ER@HL-D in hypoxic conditions (NIPP/ER@HL-D + Nitroreductase (NTR)) was 84.24% at 8 h, which was significant rapid than that of 51.67% in normal conditions (NIPP/ER@HL-D + PBS), indicating that NIPP/ER@HL-D had a good hypoxic response release profile *in vitro* (**Figure 2H**). Furthermore, compared with free DiR fluorescence, and DOTAP@HL-D, the accumulation of NIPP@HL-D was significantly enhanced in the ischemic hemisphere, indicating the hypoxic response profiles of NIPP@HL-D *in vivo*. Consequently, hypoxia-responsive liposomes fused with the neutrophil-like cell membrane demonstrated superior anti-ischemic stroke efficacy with reduced brain infarct volumes compared to their non-fused counterparts.

Calixarene-grafted peptide-based hypoxia-responsive hydrogel-loaded with fingolimod (FTY720) 129 or glucose-modified azocalixarene-based hypoxia-responsive supramolecular drug delivery system-loaded with Lip 131 could respond to hypoxic environments by reduction of azo groups, trigging the decomplexation of therapeutic drugs. While, 2-nitroimidazole derivatives-based hypoxia-sensitive liposomes-loaded with edaravone also performed potent efficacy against ischemic stroke 130. Collectively, these findings demonstrated that nanosystems with different preparations levaraging azo or nitroimidazole-based compounds for hypoxia-responsive delivery of various therapeutic drugs such as neuroprotective agent and ferroptosis inhibitor all be designed and applied for ischemic stroke, indicating that hypoxia-responsive drug delivery strategies hold significant promise for the treatment of ischemic stroke.

### 5.2. ROS-sensitive delivery systems

Ischemic stroke is characterized by elevated ROS levels within the affected brain tissue. These elevated ROS levels can mediate the release of inflammatory cytokines from endothelial cells, activate platelets, and ultimately contribute to thrombus formation 29. Paradoxically, this upregulation of ROS can also be exploited as an endogenous trigger for controlling the release of therapeutic drugs. Inspired by the accumulation of ROS in ischemic brain tissue, multifunctional ROS-responsive nanomedicines have been developed. These nanomedicines offer targeted delivery and controlled drug release within the ischemic penumbra, providing a promising strategy for ischemic stroke treatment.

**Figure 3:**
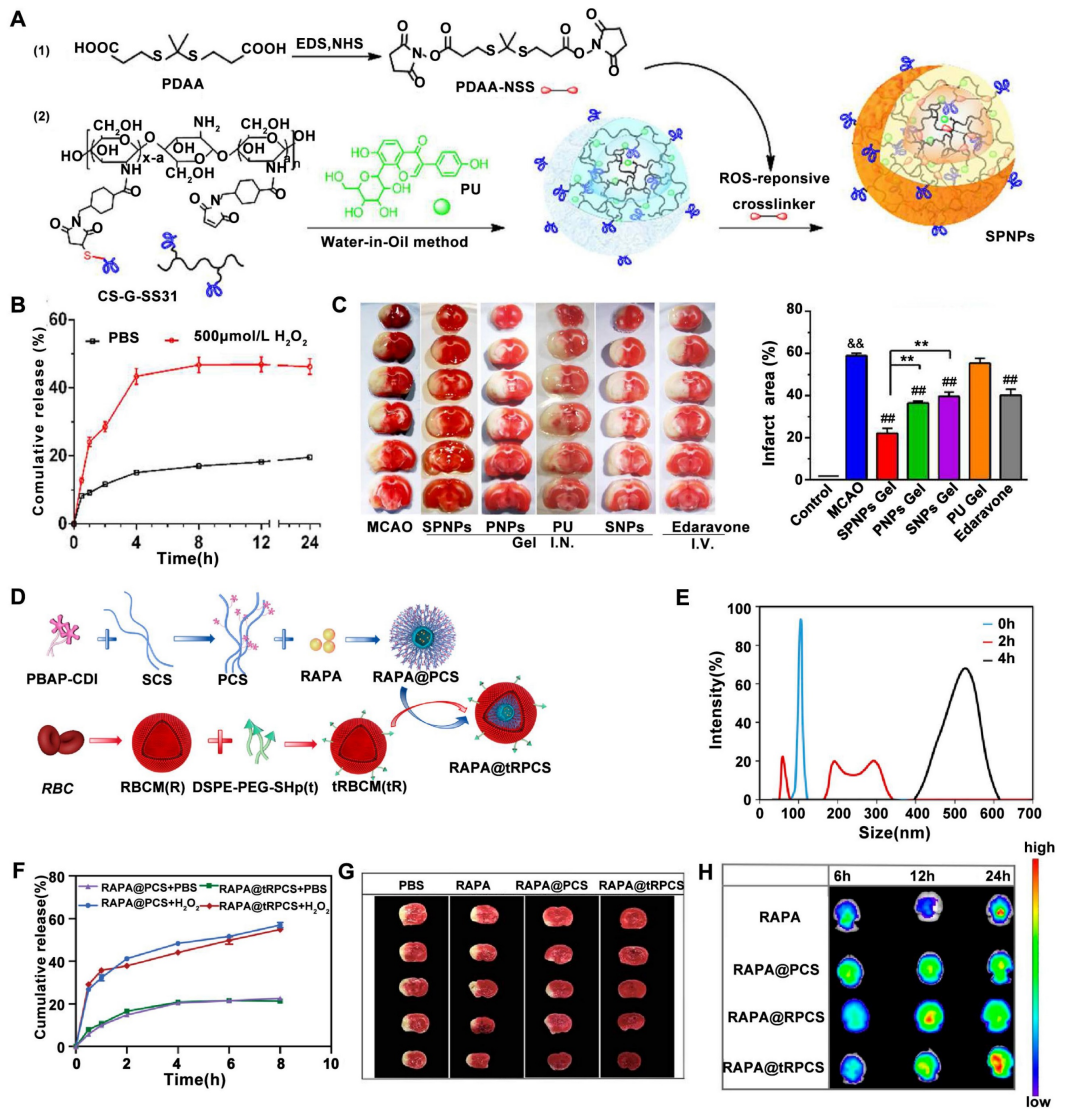
** ROS-sensitive nanoparticles for achieving specific targeted delivery and controlled drug release at the ischemic penumbra.** (A) Schematic illustration of the fabrication of ROS-responsive and mitochondria-targeted nanoparticles encapsulated with PU. (B) *In vitro* release profiles of PU from SPNPs in PBS or H_2_O_2_. (C)Triphenyltetrazolium chloride (TTC) staining images under different treatment and quantitative analysis of the ratio of infarct volume to total brain volume. Adapted with permission from 132, copyright 2023 Elsevier. (D) Schematic illustration of the preparation of RAPA@tRPCS. (E) Size Change of PCS NPs treated with H_2_O_2_ at 37 °C. (F) *In vitro* release profiles of RAPA in RAPA@PCS and RAPA@tRPCS incubated with or without H_2_O_2_ at 37℃ (n = 3). (G) Representative TTC staining pictures of brain slices with different treatments. (H) *In vivo* imaging system image of DIR fluorescence-labeled RAPA, RAPA@PCS, RAPA@RPCS, and RAPA@tRPCS in the ischemic brain *ex vivo* at 6, 12, and 24 h, respectively. Adapted with permission from 138, copyright 2024 American Chemical Society.

Recently, a novel multifunctional nanoparticle termed Self-assembled Polymeric Nanoparticles (SPNPs) with both mitochondrial targeting and ROS responsiveness has been designed 132. These SPNPs incorporate a thioketal linker and puerarin (PU) as the ROS-responsive component and the active agent for ROS depletion, respectively (**Figure 3A**). The SPNP-loaded thermosensitive hydrogel was designed for targeted brain delivery via the nose-to-brain pathway and ROS-sensitive release of the therapeutic agents. The ROS-responsive drug release of SPNP was evaluated under H₂O₂ treatment. In detail, the cumulative release of PU in the presence of H_2_O_2_ is about 48% at 8 h, but only 18% in the absence of H_2_O_2_ (**Figure 3B**). Furthermore, following administration, SPNPs-gel treatment groups significantly reduced brain infarct area in a (MCAO mouse model, demonstrating the potential of ROS-responsive nanomedicines in the treatment of ischemic stroke (**Figure 3C**). In another study, Liu *et al.* designed a ROS-responsive nanocarrier based on sulfated polysaccharides for targeted delivery of rapamycin (RAPA), a neuroprotective agent, to mitigate ischemic brain damage 138. This nanocarrier utilized a boronic ester moiety as the ROS-responsive component to facilitate the release of RAPA upon encountering high intracellular ROS levels (**Figure 3D**). The released RAPA then promoted the phenotypic shift of microglia from M1 to M2 and stimulated angiogenesis. The ROS responsiveness disintegration and the release of the nanoparticles was investigated using H_2_O_2_. Theboronic ester conjugated with SCS (PCS) NPs co-inbucation with H_2_O_2_ for 2 hours exhibited an unstable size distribution, with complete enlargement observed at 4 h (**Figure 3E**). Moreover, both of H_2_O_2_ treated RAPA@PCS and RAPA@tRPCS showed higher drug release rates than their PBS treated counterparts, respectively (**Figure 3F**). Furthermore, compared to both the RAPA-conjugated polysaccharide carrier (RAPA@PCS) and the free RAPA groups, RAPA-conjugated ROS-responsive polysaccharide carrier (RAPA@tRPCS) significantly reduced brain infarct area in a temporary middle cerebral artery occlusion (tMCAO) mouse model, highlighting the potential of ROS-responsive nanomedicines for ischemic stroke treatment (**Figure 3G**). *In vivo* imaging confirmed the successful targeting of RAPA@tRPCS to the ischemic infarct, as demonstrated by the significantly higher accumulation on the ischemic side compared to the non-ischemic side (**Figure 3H**).

**Figure 4:**
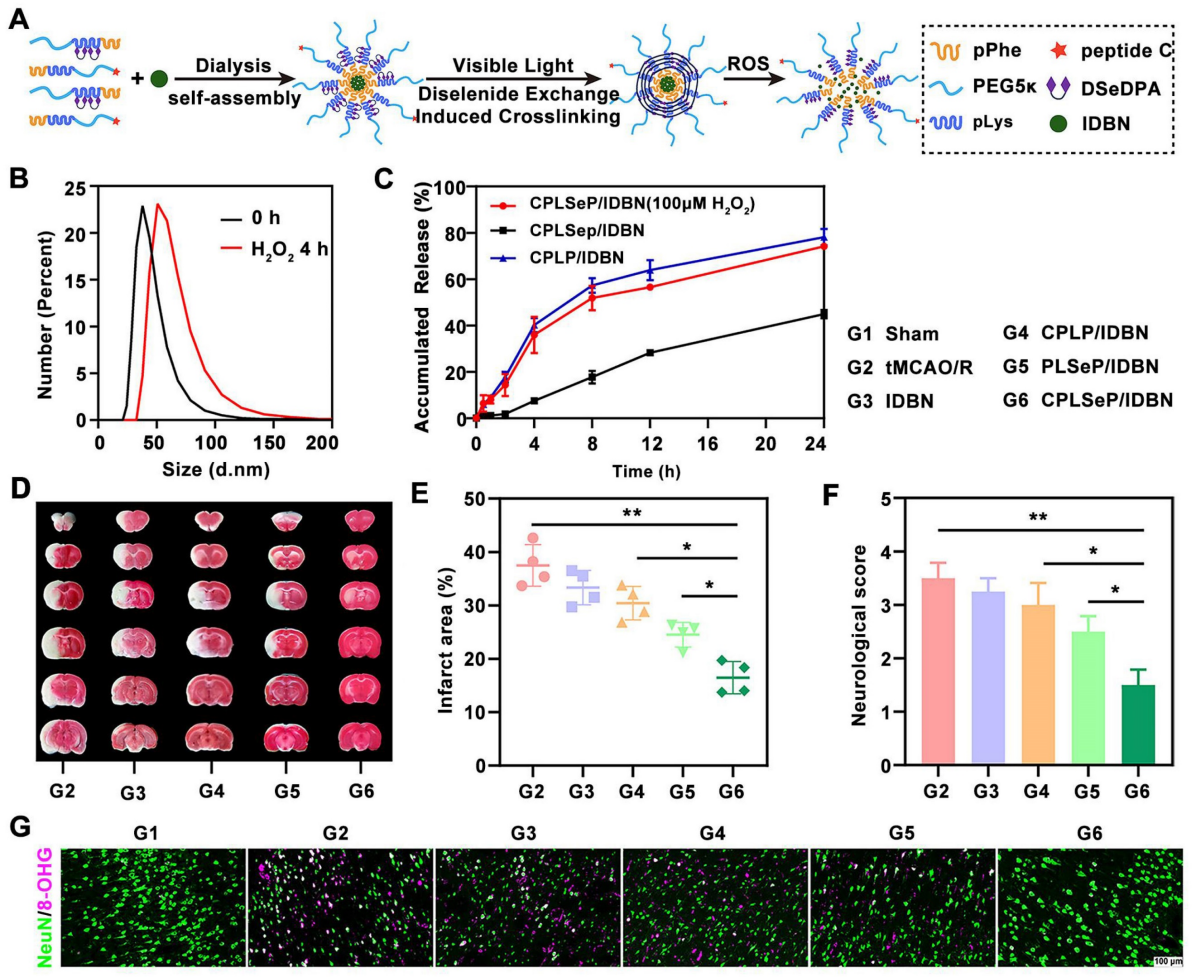
** ROS-sensitive micells for ischemic stroke treatment by micells physicochemical properties change-induced enhanced delivery of IDBN to ischemic brain tissue upon ROS trigger.** (A) Illustration of the preparation process of IDBN-loaded diselenide bond cross-linked micelles (CPLSeP/IDBN) by dialysis and dynamic diselenide exchange induced cross-linking. (B) Changes in the size distribution of CPLSeP/IDBN micelles after incubation with H2O2. (C) *In vitro* release profiles of IDBN from CPLSeP/IDBN and CPLP/IDBN micelles in Dulbecco's phosphate-buffered saline (DPBS) (pH 7.4) with or without H2O2, n = 3. (D) The Brain slices of tMCAO/R rats with different treatments stained with TTC 24 h post reperfusion. (E) The quantified results of TTC staining in (D). (F) The neurological assessment of tMCAO/R rats treated with different formulations. (G) Representative immunofluorescence staining images indicating the number of neurons and the level of DNA/RNA damage (8-OHG) in the penumbra of tMCAO/R rats with different treatments (scale bar, 100 μm). Adapted with permission from 142, copyright 2023 American Chemical Society.

It has been reported that a ROS-responsive polymeric micelle loaded with idebenone (IDBN) could target the ischemic penumbra and promote recovery by inhibiting glial overactivation and neuronal ferroptosis 142. These polymeric micelles were assembled from ROS-responsive materials, incorporating diselenide bonds that transform into hydrophilic seleninic acids within environments with high ROS accumulation. This transformation enables ROS consumption and triggers the release of the therapeutic drug (**Figure 4A**). The ROS-responsive drug release was evaluated using H₂O₂ treatment. Co-incubation with H₂O₂ resulted in an increase in the size (**Figure 4B**) and drug release rate (**Figure 4C**) of the CPLSeP/IDBN micelles. Moreover, triphenyltetrazolium chloride (TTC) staining revealed a significant reduction in infarct volume in the group treated with IDBN-loaded micelles compared to groups treated with saline or free IDBN (**Figure 4D**). The infarct volume was statistically reduced to 15% of the total brain volume (**Figure 4E**), which was further corroborated by neurological assessments in tMCAO/R rats (**Figure 4F**). Immunofluorescence staining further revealed increased neuronal survival following treatment with CPLSeP/IDBN micelles (**Figure 4G**).

To enhance the delivery efficiency of ROS-responsive nano delivery systems, intranasal administration has been applied to bypass BBB 132. Moreover, different materials such as stroke homing peptide 138, mitochondrial-targeting peptide 132 or microthrombus-binding peptide 142 have been added to ROS-responsive delivery systems to augment their selectivity. Collectively, although with a great promise, the BBB and cell/organelle selectivity remain big challenges for ROS-responsive nano delivery systems, which deserves more attention and research in the future.

### 5.3. Inflammation-sensitive delivery systems

Drawing inspiration from the natural recruitment of peripheral cells to the ischemic brain tissue, researchers have developed immune cell membrane-encapsulated nanoparticles as a novel drug delivery system for ischemic stroke treatment. This approach combines the versatile engineering capabilities of synthetic nanomaterials with the unique functionalities of cellular membranes to effectively deliver therapeutic agents. The cell membranes for these nanoparticles can be derived from various specific cells, including neutrophils, platelets, monocytes/macrophages 168 and vascular endothelial cell 169. The synthetic cores typically fall into categories such as micelles, polymeric nanoparticles, liposomes, and metal nanoparticles. These biomimetic nanosystems can interact with the complex pathological microenvironments present after a stroke, facilitating the penetration of therapeutics across the BBB for targeted drug delivery to the ischemic brain tissue 170, 171. Besides, neutrophil membrane-coated nanoagents or monocyte cell membrane-camouflaged nanoparticles can target specific pathways involved in neutrophil or monocyte infiltration into the injury site, thereby mitigating the inflammatory microenvironment.

**Figure 5:**
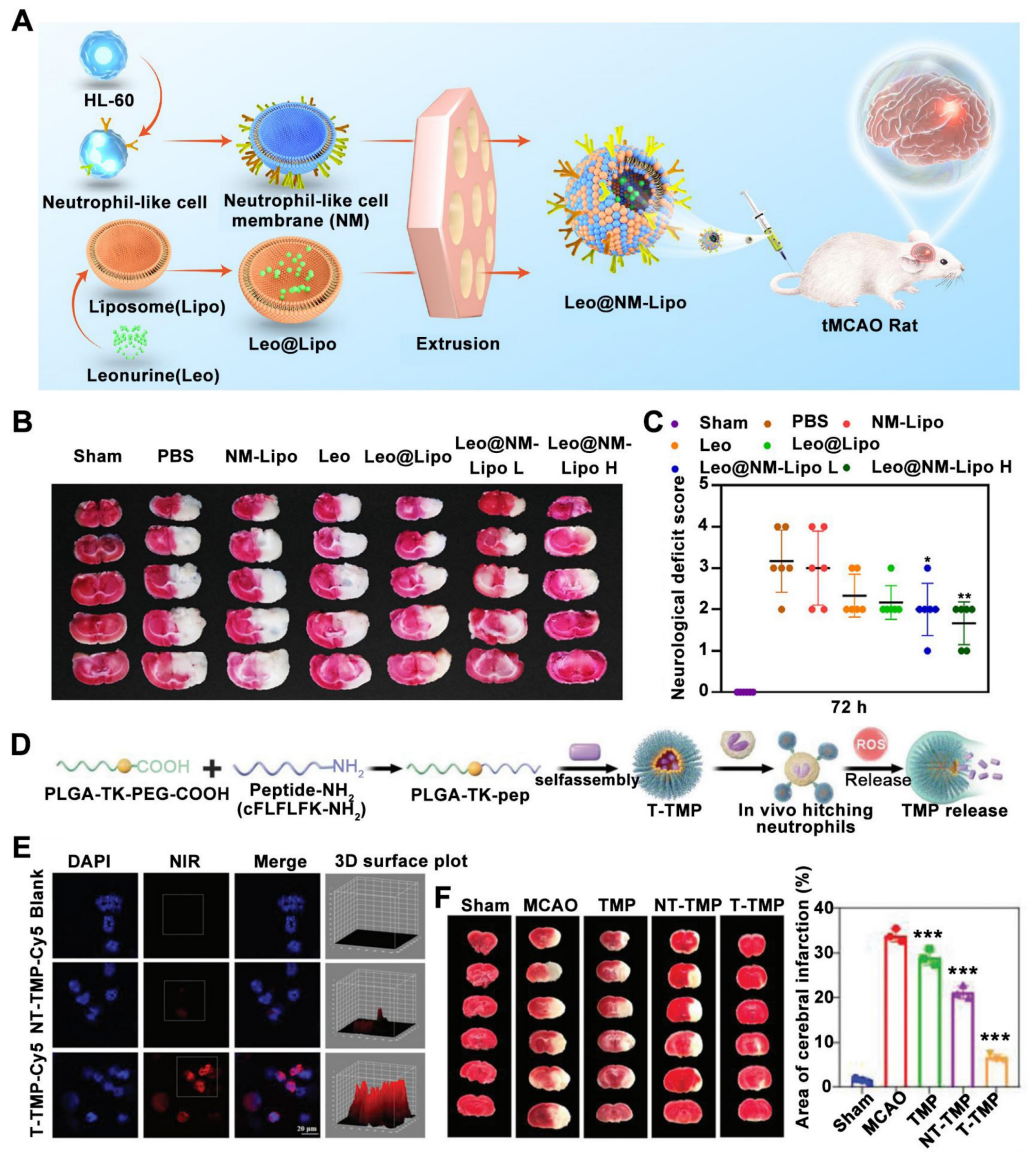
** Neutrophil recruitment-inspired nanomedicnes for ischemic stroke treatment.** (A) Illustration of a neutrophil-based biomimetic nanoplatform against ischemic stroke. (B) Representative images of TTC-stained brain slices. (C) Neurological scores in different groups. Adapted with permission from 105, copyright 2023 Elsevier. (D) Schematic of the preparation of neutrophil-targeted and ROS-reactive TMP nanoparticles (T-TMP). (E) Confocal images of Cy5-labeled T-TMP and NT-TMP uptake by activated neutrophils *in vitro*. (F) TTC staining pictures of brain slices with different treatments. All images were reproduced with permission. Adapted with permission from 43, copyright 2023 John Wiley and Sons.

Wang *et al.* recently described a neutrophil-mimicking nanoplatform for intracerebral drug delivery in ischemic stroke. This platform was designed by coating human promyelocytic leukemia cell-derived membranes (NM) onto leonurine (Leo)-loaded nanoliposomes (Lipo) (**Figure 5A**) 105. Leo@NM-Lipo targets the ischemic lesion and rescues the injured brain by alleviating neuronal apoptosis, oxidative stress, neuroinflammation, and restoring BBB integrity in tMCAO rats. TTC staining revealed that Leo@NM-Lipo H demonstrably reduced the infarct area, as evidenced by the diminished white-colored region in the ischemic hemisphere (**Figure 5B**). Moreover, Leo@NM-Lipo H treatment resulted in a significant improvement in neurological deficits compared to the model group (**Figure 5C**).

In addition to neutrophil membrane-coated nanoparticles for ischemic stroke treatment, nanoparticles hitchhiking on neutrophils for the enhanced treatment of cerebral ischemia-reperfusion injury have been reported more recently 43. This nanoparticle is obtained by self-assemble of ligustrazine (TMP) and a novel amphiphilic polymer containing ROS cleavable ketoaldehyde and neutrophil formyl peptide receptors-targeted peptide (**Figure 5D**). By co-incubating activated neutrophils with indendopolysaccharide cyanocyanide (Cy5)-labeled NPs, it was observed under confocal images that neutrophils in the T-TMP-treated group exhibited stronger red fluorescence compared to the NT-TMP group and blank group, indicating that T-TMP exhibited higher neutrophil uptake/ hitchhiking (**Figure 5E**). Furthermore, regarding therapeutic efficacy, compared to the T-TMP group 3 days after MCAO, T-TMP treated animals distinctly reduced the infarct area (**Figure 5F**).

**Figure 6:**
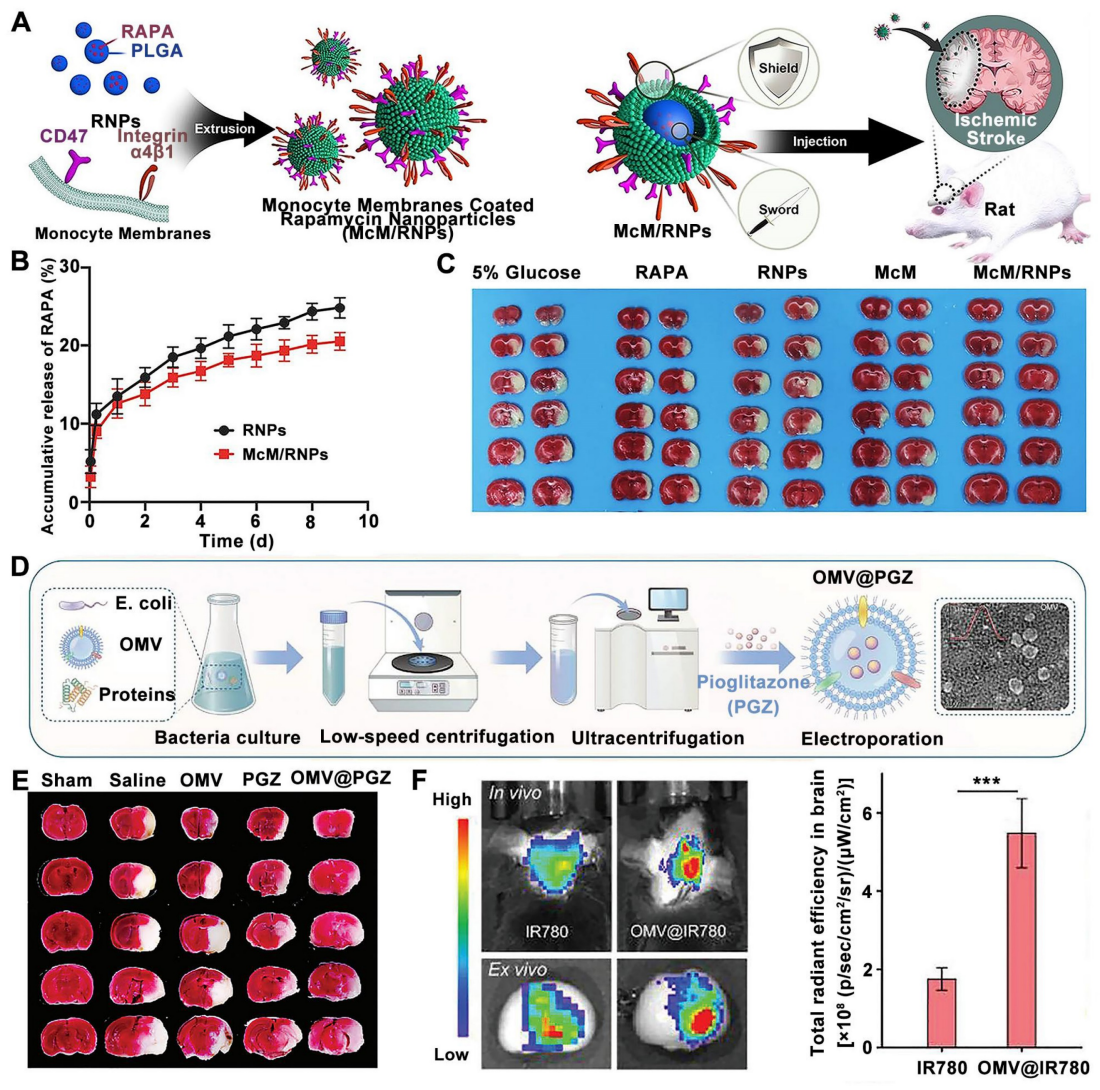
** Monocyte cell membrane and bacteria-derived outer-membrane biomimetic nanosystems for ischemic stroke treatment.** (A) Schematic diagram the fabrication and application of McM/RNPs for ischemic stroke. (B) *In vitro* release curves of RNPs and McM/RNPs. (C) TTC staining pictures of brain slices with different treatments. Adapted with permission from 106, copyright 2021 BioMed Central. (D) Schematic of ischemic stroke mediated by OMV@PGZ nanoparticles. (E) Representative pictures of TTC staining in brain sections and quantification of the infarct size of the ischemic brain hemisphere. (F) Accumulation and Semi-quantification of OMV 24 h post-injection in the ischemia-affected brain *in vivo*. Adapted with permission from 108, copyright 2023 John Wiley and Sons.

In addition to neutrophil-inspired nanosystmes, RAPA-loaded nanoparticles coated with monocyte membrane (McM) have been designed for attenuating inflammation and ischemia/reperfusion injury by blocking monocyte infiltration and inhibiting microglia proliferation 106. In detail, the monocyte membrane biomimetic nanosystems were prepared by coating monocyte-derived membranes onto preformed poly (lactic-co-glycolic acid) cores loaded with RAPA, named McM/RNPs to achieve synergistic immunotherapy (**Figure 6A**). Compared to RNPs, the RAPA release profiling of McM/RNPs is slightly slower, suggesting that their potential to be used for sustained drug release (**Figure 6B**). Moreover, the cerebral infarction lesions were significantly improved in MCM/RNPs treated rats compared with other treatment groups (**Figure 6C**). In another approach, bacteria-derived outer-membrane vesicles (OMVs) have been explored for enhanced brain delivery of therapeutic agents against ischemic stroke by exploiting a "neutrophils hitchhiking" mechanism 108 (**Figure 6D**). TTC staining analysis demonstrated that among all groups, OMV@PGZ treatment resulted in a minimal infarct volume following administration (**Figure 6E**). Moreover, *ex vivo* imaging revealed a significantly stronger fluorescence signal in the infarct brain of mice treated with OMV@IR780 compared to IR780-treated mice (**Figure 6F**).

In addition to neutrophil membranes, monocyte membranes, OMV etc, platelet membrane biomimetic nanocarriers have also been applied for early ischemic stroke through in situ generation of nitric oxide by L-arginine metabolism 109. These findings suggest that inflammation-sensitive nanomedicines (leonurine 105, Ligustrazine 43) hold immense promise for targeted treatment of ischemic stroke as ongoing research continues to validate its effectiveness. Despite significant advancements in inflammation-related cell recruitment-based biomimetic nanosystems, further research such as scalability, reproducibility, storage stability, biocompatibility and validity in human patients of the biomimetic nanosystems are urgently needed to fully exploit the capabilities of these systems for ischemic stroke treatment.

### 5.4. pH-sensitive delivery systems

Ischemic stroke disrupts normal brain function, leading to metabolic acidosis within the affected tissue. This phenomenon, caused by factors such as anaerobic glycolysis, results in a lower pH microenvironment compared to healthy brain tissue 172. This unique pH gradient between ischemic and normal tissues presents an opportunity to develop pH-responsive drug delivery systems. These systems can ensure the targeted release of therapeutic agents, specifically within the ischemic region, thereby minimizing the risk of off-target side effects. In recent years, pH-responsive nanoplatforms have emerged as promising alternative strategies for ischemic stroke treatment.

**Figure 7:**
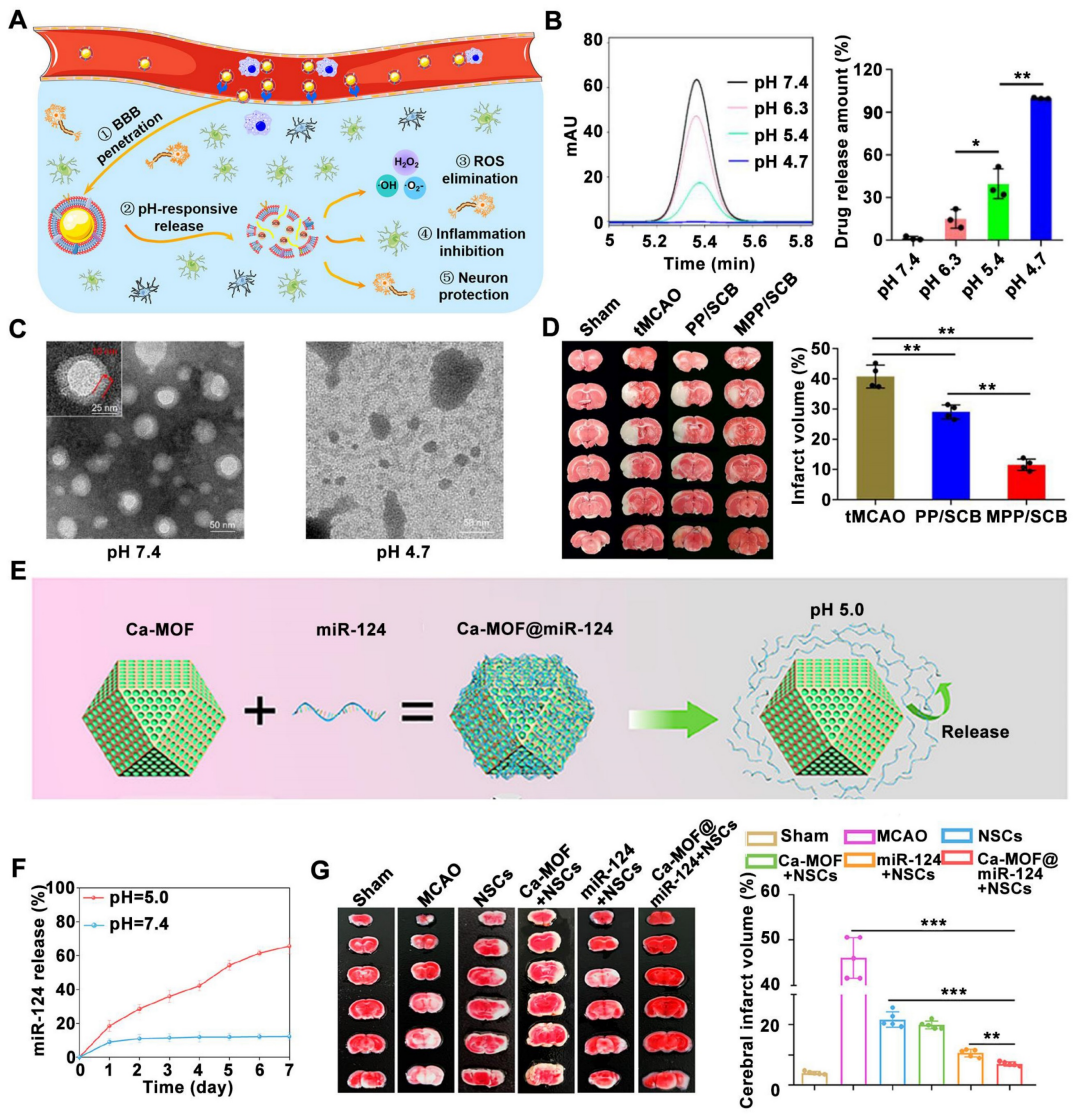
**pH-responsive nDDS for targeted drug release within ischemic regions.** (A) Illustration of the tumor cell membrane camouflaged pH-responsive nanomedicine for ischemic stroke treatment. (B) *In vitro* release of MPP/SCB in citrate buffer at different pH at 15 min. (C) TEM images of MPP/SCB in PBS at pH 7.4 or pH 4.7. (D) Representative TTC staining pictures of the brain slices in tMCAO rats with different treatments. Adapted with permission from 146, copyright 2021 American Chemical Society. (E) Schematic of the conjugation and the rapid cleavage of the hydrogen bonds between miR-124 and the surface of Ca-MOF upon exposure to an acidic environment. (F) Release curves of miR-124 from Ca-MOF@miR-124 nanoparticles incubated in PBS (pH 7.4) or Tris-HCl (pH 5.0). (G) Representative TTC-stained images and quantitative analysis of the infarct volume. Adapted with permission from 173, copyright 2023 American Chemical Society.

Inspired by the invasive behavior of 4T1 cancer cells during brain metastasis, Sha *et al.* developed a biomimetic nanoplatform by camouflaging a succinobucol (SCB)-loaded pH-sensitive polymeric nano vehicle with a 4T1 cell membrane (MPP/SCB) 146. MPP/SCB demonstrates superior BBB penetration and preferential accumulation within the ischemic brain region. Under acidic conditions, characteristic of the ischemic environment, the platform triggers the release of therapeutic agents, leading to therapeutic effects (**Figure 7A**). SCB was barely released at pH 7.4, but significantly released in acidic environments was observed (**Figure 7B**), confirming the pH-responsive of SCB. Meanwhile, compared to the Transmission electron microscope (TEM) images at pH 7.4, the typical spherical structures of MPP/SCB were difficult to observe at pH 4.7 (**Figure 7C**), confirming the pH-responsiveness of MPP/SCB. Treatment with MPP/SCB resulted in a significant reduction in infarct volume within the ischemic hemisphere (**Figure 7D**), highlighting the potential of pH-responsive nanomedicines for ischemic stroke therapy. In another study, Liu *et al.* designed miR-124-loaded calcium metal-organic framework (Ca-MOF) nanoparticles in which neuron-specific miR-124 can promote the differentiation of NSCs into mature neurons for ischemic stroke treatment 173. The surface of these Ca-MOF nanoparticles facilitates the conjugation of miR-124 via hydrogen bonds. In an acidic environment, such as the one present after a stroke, these hydrogen bonds are rapidly broken, triggering the release of miR-124 (**Figure 7E**).

When the Ca-MOF@miR-124 nanoparticles were incubated in PBS (pH 7.4), only a small amount of miR-124 was released on the first day, after which no significant changes were observed; However, when incubated in Tris-HCL (pH 5.0), a sustained release of miR-124 is observed, confirming the pH-responsiveness of Ca-MOF@miR-124 nanoparticles (**Figure 7F**). The combination therapy using NSCs and miR-124-loaded Ca-MOF nanoparticles resulted in the smallest cerebral infarct volume among all groups subjected to MCAO surgery (**Figure 7G**).

**Figure 8:**
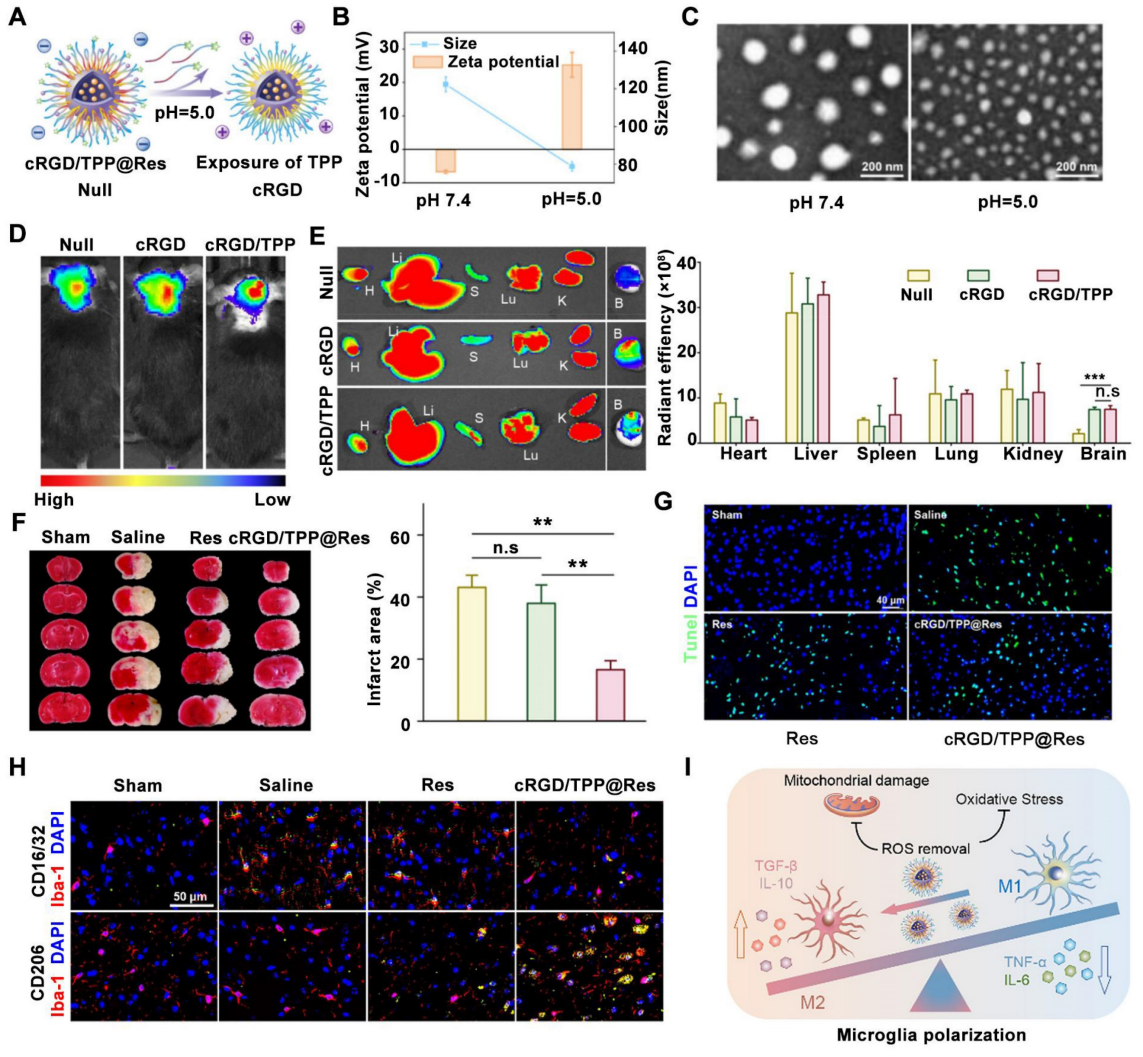
** pH-sensitive nanomedicines for ischemic stroke treatment by micellar physicochemical properties change-induced enhanced ischemic brain tissue delivery of Res (resveratrol, Res) upon an acid trigger.** (A) Schematic of TPP exposure at pH 5.0. (B, C) Zeta potential, particle size, (B) and morphology (C) changes of cRGD/TPP micelles upon acid trigger. (D) Accumulation of cRGD/TPP *in vivo* after intravenous injection. (E) *Ex vivo* fluorescence imaging and average radiation efficiency of major organs in each group. (F) Typical TTC staining pictures and quantitative analysis of cerebral infarct size. (G) Representative TUNEL staining pictures of the ischemic brain with different treatments. (H) Schematic illustration of the ratio modulation of M1 and M2 microglia for anti-inflammatory and antioxidative effects. (I) Typical immunofluorescence staining pictures of M1 microglia and M2 microglia in different groups. Adapted with permission from 161, copyright 2023 American Chemical Society.

A novel hypoxia-responsive shell-sheddable cRGD/TPP@Res micelle system has been constructed for ischemic stroke treatment to alleviate oxidative stress and inflammation 161. These micelles are composed of pH-responsive materials modified with triphenylphosphine (TPP) and cRGD peptide. TPP is conjugated to a short polyethylene glycol (PEG) chain, while cRGD is linked to a longer PEG chain. Within lysosomes, the acidic environment triggers the detachment of the long PEG shell, exposing the TPP group. This process results in a shift in the zeta potential of the micelles from negative to positive (**Figure 8A**). Furthermore, the cleavage of an acetal linkage in the acidic environment leads to a decrease gradually from 121.9 to 78.8 nm in the average size of the nanoparticles, as confirmed by both dynamic light scattering (**Figure 8B**) and TEM (**Figure 8C**). The exposure of the TPP group upon acetal cleavage also contributes to the positive zeta potential shift observed in cRGD/TPP@Res (**Figure 8B**). *In vivo* imaging using an *In Vivo* Imaging System system was employed to evaluate the delivery efficiency of cRGD/TPP@Res compared to cRGD/TPP and RGD micelles following intravenous injection in tMCAO mice. The results demonstrated a higher accumulation of both cRGD/TPP and RGD micelles in the ischemic brain compared to the control group (Null) (**Figure 8D**). Notably, a significant difference in fluorescence intensity was observed between the targeted (cRGD/TPP) and non-targeted (RGD) groups within the brain tissue (**Figure 8E**), highlighting the targeting efficacy of the cRGD moiety. Regarding therapeutic effects, cRGD/TPP@Res treatment led to a 61.5% reduction in infarct volume compared to untreated tMCAO mice (**Figure 8F**). TUNEL staining revealed a significant decrease in apoptotic cells following cRGD/TPP@Res treatment compared to treatment with Res alone, as indicated by reduced TUNEL fluorescence in the brain tissue (**Figure 8G**). Immunofluorescence staining confirmed these findings, demonstrating an increase in CD206-positive M2 microglia and a decrease in CD16/32-positive M1 microglia within the damaged brain tissue of cRGD/TPP@Res-treated mice compared to Res-treated mice (**Figure 8H**). Mechanistically, these micelles could promote the targeted delivery of resveratrol to microglial mitochondria, effectively alleviating neuroinflammation and facilitating the phenotypic shift of microglia from the pro-inflammatory M1 state to the anti-inflammatory M2 state (**Figure 8I**). These findings suggest that pH-responsive multifunctional nanoparticles are promising delivery systems for achieving accurate diagnosis and treatment of ischemic stroke *in vivo*.

## 6. Conclusions and perspectives

Ischemic stroke, a medical emergency resulting from reduced blood flow to a specific brain region, presents a significant clinical challenge. The pathophysiology of ischemic stroke involves a complex interplay of factors, including insufficient oxygen and energy availability, neuroinflammation, a decrease in pH due to the switch from aerobic to anaerobic glycolysis, oxidative stress caused by ROS generation, and disruption of the BBB. Current treatment approaches for ischemic stroke, primarily focused on vascular recanalization and neuroprotection 174, 175, often fall short of meeting the urgent needs of patients. Therefore, the development of novel therapeutic strategies is critical. In recent years, there has been a surge of interest in nanoparticle-based drug delivery systems designed to target the specific pathological microenvironment of ischemic stroke 11, 176. These systems, responsive to hypoxia, ROS, inflammation, and pH changes, can enhance drug accumulation and control the release of neuroprotective agents and/or thrombolytic drugs within the ischemic brain. The application of nanomedicine, particularly stimuli-responsive nanomedicine, for ischemic stroke therapy is gaining traction 129, 177, 178 and holds promise as a future alternative to traditional treatment strategies.

Despite the promising clinical potential and rapid development of nanomedicine and stimuli-responsive nanomedicines for ischemic stroke therapy, several challenges must be addressed before widespread clinical application. Firstly, the ischemic brain microenvironment is dynamic, characterized by changes in hypoxia, oxidative stress, inflammation, and pH over time. To effectively combat these dynamic changes, future advancements should focus on designing nano-systems that can exert a protective effect in a spatiotemporal manner. Furthermore, research efforts should prioritize the development of nanoparticles that can target specific brain cells and even organelles, tailoring delivery to the specific therapeutic agent being used. Secondly, current therapeutic strategies often target one or two mechanisms of ischemic stroke 179-181, failing to provide comprehensive protection due to the disease's complex pathophysiology. A significant challenge lies in the lack of effective drug delivery strategies to target the multiple pathological cascades involved in ischemic stroke. Co-delivery of different therapeutics using these nanocarriers presents a promising strategy for multifaceted protection. For example, neuroprotective agents can extend the time window for successful thrombolysis 182. Thus, combining nanomedicines with traditional therapies holds significant promise for the future of stroke treatment. As for targeted delivery systems, thrombosis or hemodynamic-responsive nanosystems have been developed to trigger controllable drug delivery and release in the brain tissue for ischemic stroke treatment 139, 183. Moreover, Enzyme-responsive nanosystems such as thrombin-responsive nanosystems 184 and matrix metalloproteinase-responsive nanosystems 185 have been developed for ischemic stroke therapy. Therefore, future research efforts should focus on developing novel multi-stimuli-responsive carrier systems capable of co-encapsulating different drugs that target both neuroprotection and thrombolysis while minimizing side effects.

Despite substantial scientific progress in nanomedicine for ischemic stroke, a significant gap exists between promising preclinical research and successful clinical translation. This disparity can be attributed to several key factors. Firstly, the commonly used MCAO model in animal studies often fails to fully replicate the complex pathophysiology observed in human stroke patients. Consequently, therapeutic efficacy demonstrated in these models may not translate directly to patients. The use of larger animal models, such as rhesus monkeys that more closely mimic the human ischemic microenvironment is warranted for the evaluation of ischemic stroke therapies, particularly those involving microenvironment-responsive nanomedicines. Secondly, the lack of well-defined and validated endpoint biomarkers for ischemic stroke poses a significant challenge. These biomarkers are crucial for guiding treatment decisions, predicting patient response to therapy, and optimizing personalized treatment approaches. Thirdly, current safety and biocompatibility assessments of stimuli-responsive nanomedicines may be insufficient to ensure their safe clinical application. While nanomedicines have yet to achieve widespread clinical use for ischemic stroke treatment, the future holds promise. Developing individualized treatment strategies based on microenvironment-responsive nanomedicines tailored to specific patient populations is expected to accelerate their clinical translation. While the concept of microenvironment-responsive nanomedicines for ischemic stroke therapy holds promise, the field remains in its early stages of development. Extensive research is necessary to bridge the gap between preclinical proof-of-concept and successful clinical translation. In-depth studies are urgently needed to elucidate the actual drug delivery efficiency of these nanomedicines to ischemic brain tissue. A deeper understanding of the factors influencing successful clinical translation is also critical. Without addressing these knowledge gaps, the clinical application of microenvironment-responsive nanomedicines for ischemic stroke will remain hindered. Despite these challenges, stimuli-responsive nanomedicines represent a promising avenue for future stroke treatment strategies.

## Figures and Tables

**Table 1:** Representative hypoxia-sensitive elements for ischemic stroke-microenvironment-responsive nano-systems.

Hypoxic-responsive materials	Hypoxic-responsive mechanism	Hypoxic-responsive enzymes	References
Nitroimidazole	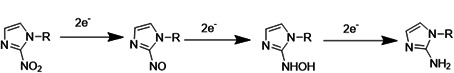	Nitroreductase (NTR); Nitroreductase	60, 61
Azobenzene	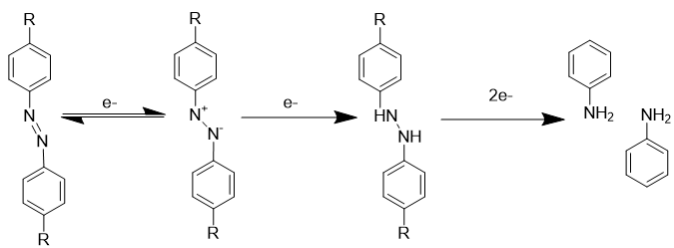		62, 63
Nitrobenzyl alcohol	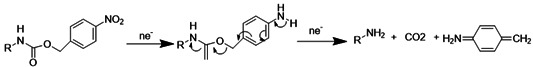		64, 65

**Table 2:** Representative ROS-sensitive elements for ischemic stroke-microenvironment-responsive nano-systems.

ROS-responsive materials	ROS-responsive mechanism	References
Thioketa	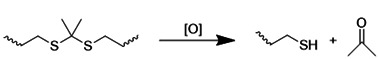	75, 76
Chalcogen thioether	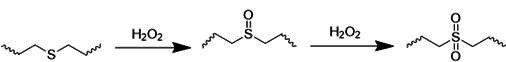	77, 78
Selenide	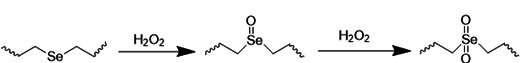	79, 80
Telluride	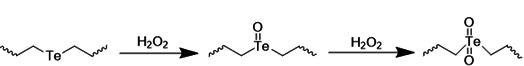	81, 82
Diselenide	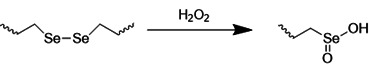	83, 84
Peroxalate ester	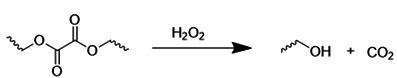	85
Arylboronic ester	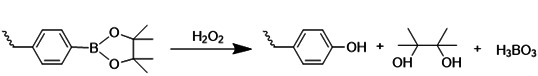	86, 87
Vinyldithioether	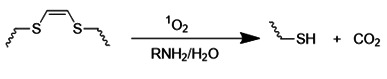	88, 89
Polypropylene sulfide	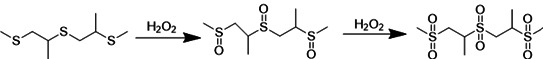	90, 91
Aminoacrylate	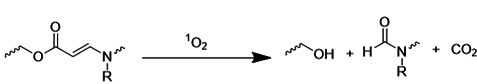	92, 93
Oligoproline	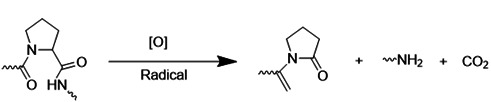	94

**Table 3:** Representative inflammation-sensitive elements for ischemic stroke-microenvironment-responsive nano-systems.

Cell Membrane Source	Cell Membrane Feature	Function	Reference
Neutrophil	Penetration of the BBB, Targeting of infarct core, Specific targeting of inflammatory tissue, Endothelial adhesion, Immune evasion properties of neutrophils	Facilitating nanoparticles to access the damaged brain via targeting to inflamed brain microvascular endothelial cells	103-105
Monocyte	Targeting and binding to inflammatory endothelial cells, Inhibiting the adhesion of monocytes to the endothelium and endothelial adhesion	Achieving synergistic immunochemotherapy therapy of ischemic stroke	106
Macrophage	Antigen recognition, Targeting the site of cerebral ischemia and inflamed tissues	Regulating neuroinflammation	107
Bacteria	Promoting adaptive immunity, Recognized by neutrophil Toll-like receptors	Facilitating the endocytosis of vesicles by neutrophils, neutrophil hitchhiking-mediated brain targeting nanoplatform	108
Platelet	Targeting adhesion to the damaged blood vessel, Adherence to inflammatory neutrophil	Targeting of ischemic stroke lesions area and the treatment of ischemic stroke	109, 110

**Table 4:** Representative pH-sensitive elements for ischemic stroke-microenvironment-responsive nano-systems.

pH-responsive materials	pH-responsive mechanism	References
Hydrazone bond	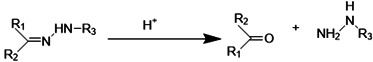	113-115
Imine bond	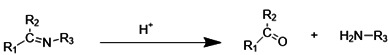	116-118
Oxime bond	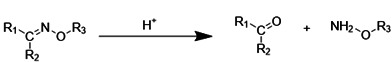	119, 120
Ortho ester bond	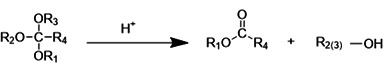	121-123
Borate ester bond	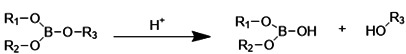	124, 125
β-thiopropionate	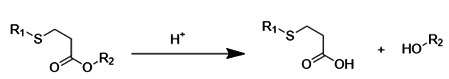	126-128

**Table 5:** Summary of microenvironment-responsive nano delivery systems for ischemic stroke.

Stimulus	Nanocarriers	Responsive elements	Immunotherapeutic drug	Therapeutic efficacy	Source
Hypoxia	Hydrogel	Azobenzene	CY5-DM/ FTY720	Treatment of ischemic stroke Improving inflammation	129
	Liposomal	2-Nitroimidazole	HYD	Increasing BBB permeability Improving neuronal apoptosis	130
	Macrocyclic carrier	Azobenzene	Lip	protecting the BBB reducing ferroptosis	131
ROS	Nanoparticle	Thioketal bond	TMP	Improving inflammation	43
	Nanoparticle	Thioketal bond	PU	Decreasing oxidative stress Improving inflammation Improving neuronal apoptosis	132
	Liposomal	Thioketal bond	CI-amidine	Improving inflammation Maintaining the integrity of the BBB Protecting of neuronal function	133
	Micelle	Thioketal bond	Luteolin	Improving inflammation Decreasing oxidative stress	134
	Nanoparticle	Thioketal bond	DSS	Improving inflammation Decreasing oxidative stress Improving neuronal apoptosis	135
	Liposomal	Thioketal bond	HET0016	Improving inflammation Improving neurological deficit	136
	Polymeric nanoparticle	Boronic ester	GA	Improving inflammation	137
	Nanoparticle	Boronic ester	RAPA	Promoting angiogenesis Improving inflammation	138
	Micelle	Phenylboronic ester	RAPA	Improving inflammation Decreasing oxidative stress	139
	Nanoparticle	PBAP	Ac2-26	Decreasing oxidative stress Improving inflammation Improving neuronal apoptosis	140
	Nanoparticle	PBAP	NBP	Decreasing oxidative stress Improving inflammation Improving neuronal apoptosis	141
	Micelle	Diselenide bond	IDBN	Improving neuronal apoptosis Improving inflammation	142
	Nanoparticle	Diselenide bond	DHA	Improving inflammation Improving neuronal apoptosis	143
	Nanoparticle	Oxalate bond	PAPA	Decreasing oxidative stress Improving inflammation	144
	Nanoparticle	Oxalate bond	Cur	Decreasing oxidative stress Improving inflammation Improving neuronal apoptosis	145
Inflammation	Nanoparticle	Bacteria membrane	PGZ	Improving inflammation Inhibiting ferroptosis	108
	Nanoparticle	Cancer membrane	SCB	Decreasing oxidative stress Improving inflammation	146
	Liposomal	Macrophage membrane	PNS and Rg3	Improving inflammation Improving neuronal apoptosis	107
	Nanoparticle	Platelet membrane	L-arginine andγ-Fe2O3	Enhancing angiogenesis Reducing PLT aggregation	109
	Nanobuffer	Neutrophil membrane	CBD	decreasing oxidative stress Improving inflammation Improving neuronal apoptosis	147
	Nanoparticle	Neutrophil membrane	SPIO	Improving inflammation	148
	Nanoparticle	Neutrophil membrane	RvD2	Improving inflammation	104
	Nanozyme	Neutrophil membrane	MPBzyme	Improving inflammation Improving neuronal apoptosis	149
	Nanoparticle	MSC membrane	A151	Improving inflammation Decreasing oxidative stress	150
	Nanoparticle	Monocyte membranes	RAPA	Improving inflammation	106
pH	Nanoparticle	BAM	NA1	Anti-oxidative stress	151
	Nanocapsule	EGDMA	IVIg	Improving inflammation	152
	Nanoparticle	PAA	t-PA	decreasing oxidative stress	153
	Micelle	DHASM	SDF-1α	Promoting angiogenesis	154
	Nanoparticle	PEOz	GW280264X and desmosterol	Improving inflammation	155
	Polymeric vesicles	Poly(β-amino ester)	DNase 1	Improving inflammation	156
	Nanoparticle	PDPA	RAPA	Improving the contrast signal in the lesion site accurate imaging and tracing Neuroprotective	157
	Nanoparticle	Phosphate groups	NTs	Reducing brain infarct volumes Enhancing cell survival	158
	Nanogels	Benzamide bonds	UK	Protecting the integrity of the BBB Improving inflammation Improving neuronal apoptosis and neurotoxicity	159
	Nanoparticle	Amide bonds	SAG	Promoting angiogenesis Reducing vascular permeability Improving neuroplasticity and neurological recovery	160
	Micelle	Acetal bonds	Res	Improving inflammation Decreasing oxidative stress	161
	Nanoparticle	Schiff-base bond	Diosgenin	Preventing thrombosis by inhibiting activation and apoptosis of platelet	162
	Nanoparticle	Imine bonds	Natural Polyphenols and Met	Improving inflammation Decreasing oxidative stress	163

Abbreviations: CY5-DM: Cyanine 5-dimethyl; FTY720: Fingolimod; HYD: hydroxyurea; Lip: Liproxstatin; TMP: ligustrazine; PU: puerarin; DSS: Danshensu; HET0016: N-hydroxy-N-4-butyl-2-methylphenylformamidine; GA: 18β-glycyrrhetinic acid; RAPA: rapamycine; NR2B9C: a neuroprotective agent; Ac2-26: an inflammation-resolving peptide; NBP: dl-3-n-butylphthalide; IDBN: idebenone; DHA: docosahexaenoic acid; Cur: curcumin; PGZ: pioglitazone; SCB: succinobucol; PNS: Panax notoginseng saponins; Rg3: Ginsenoside Rg3; CBD: cannabidiol; SPIO: superparamagnetic iron oxide; RvD2: Resolvin D2; MPBzyme: Mesoporous Prussian blue nanozyme; A151: cGAS inhibitor, telomerase repeat sequences; NA1: a neuroprotective peptide; IVIg: immunoglobulin; t-PA: tissue plasminogen activator; SDF-1α: Stromal cell-derived factor-1; GW280264X: an ADAM17 inhibitor; Desmosterol: an liver X receptor agonist; NTs: Nucleotides; UK: urokinase; SAG: smoothened agonist; Res: Resveratrol; Met: Metformin.
